# Diffusion Mapping of Eosinophil‐Activation State

**DOI:** 10.1002/cyto.a.23884

**Published:** 2019-08-31

**Authors:** Justyna Piasecka, Catherine A. Thornton, Paul Rees, Huw D. Summers

**Affiliations:** ^1^ Swansea University Medical School Swansea University Swansea SA2 8PP UK; ^2^ Systems and Process Engineering Centre, College of Engineering Swansea University Swansea SA1 8EN UK

**Keywords:** eosinophil, imaging flow cytometry, multivariate analysis, diffusion maps

## Abstract

Eosinophils are granular leukocytes that play a role in mediating inflammatory responses linked to infection and allergic disease. Their activation during an immune response triggers spatial reorganization and eventual cargo release from intracellular granules. Understanding this process is important in diagnosing eosinophilic disorders and in assessing treatment efficacy; however, current protocols are limited to simply quantifying the number of eosinophils within a blood sample. Given that high optical absorption and scattering by the granular structure of these cells lead to marked image features, the physical changes that occur during activation should be trackable using image analysis. Here, we present a study in which imaging flow cytometry is used to quantify eosinophil activation state, based on the extraction of 85 distinct spatial features from dark‐field images formed by light scattered orthogonally to the illuminating beam. We apply diffusion mapping, a time inference method that orders cells on a trajectory based on similar image features. Analysis of exogenous cell activation using eotaxin and endogenous activation in donor samples with elevated eosinophil counts shows that cell position along the diffusion‐path line correlates with activation level (99% confidence level). Thus, the diffusion mapping provides an activation metric for each cell. Assessment of activated and control populations using both this spatial image‐based, activation score and the integrated side‐scatter intensity shows an improved Fisher discriminant ratio *r*_d_ = 0.7 for the multivariate technique compared with an *r*_d_ = 0.47 for the traditional whole‐cell scatter metric. © 2019 The Authors. *Cytometry Part A* published by Wiley Periodicals, Inc. on behalf of International Society for Advancement of Cytometry.

## Graphical abstract

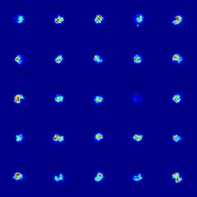

Automated analysis of single‐cell data, using multivariate approaches with imaging platforms, was first reported over 20 years ago by Beksaç et al. 1. Today, there is a growing appreciation of this approach 2 as the use of machine learning algorithms has become widespread 3, 4. However, the majority of reported studies focus at the whole cell level and the identification of the cell type 5, 6, even though this may well involve the extraction of intracellular morphology metrics 7. Here, we report on the use of diffusion mapping, a time inference algorithm to provide a trajectory of cells based on image features measuring their intracellular state 8. The aim is to profile the status of eosinophils by their granular substructure and visualize in a low‐dimensional space. Eosinophils are specialized myeloid cells that play a central role in allergy and infection; hence, better strategies for enumerating and functionally evaluating them are critical to disease diagnosis and monitoring 9. They are terminally differentiated, end‐stage cells that when fully matured contain cytoplasmic, crystalline granules, which are both storage and secretory organelles 10, 11, 12, 13. These granules have a unique ultrastructural morphology 14 and their contents are secreted upon stimulation with a number of physiological stimuli such as cytokines and chemokines 15. Release occurs by extracellular secretion of intact granules or by progressive emptying of specific granule contents in the absence of granule‐to‐granule or granule‐to‐plasma membrane fusions via a process termed piecemeal degranulation (PMD) 16, 17. This activation of eosinophils by molecular stimuli is associated with changes to the morphology and spatial distribution of the granules as they release proteins from their core matrix and are transported to the cell surface. Given the submicrometer size of these granular structures, the dynamics of eosinophil activation are typically studied by electron microscopy 18. Here, we present studies in which optical microscopy, implemented using an imaging flow cytometer, is used to obtain multiple morphological feature measurements 19. Multivariate analysis of these features provides a quantitative assessment of the eosinophil‐activation state that is determined by the optical transmission and scattering properties of the intracellular granules.

Current, commonly used blood diagnostics rely on the simple measure of eosinophil count 20, 21 to discern the presence of anomalous immune system activity 22, 23, 24. However, the activation status of the cells is intimately linked to the progress and current status of allergic and inflammatory disease 25. It is not only, therefore, the prevalence of eosinophils that is important, their functional state must also be considered. Quantification of the degree of eosinophil activation can be obtained through the use of fluorescently labeled CD‐specific antibodies 25, 26, 27. Here, we demonstrate that label‐free profiling of the activation state of each eosinophil is also possible from side‐scatter (dark‐field) images, taken with an Imagestream cytometer (Merck, Darmstadt, Germany), from peripheral blood samples obtained from healthy human donors. The analysis data are obtained by multivariate quantification of 85 image features extracted from pixel‐level measurements of cell scatter, collected orthogonal to the illumination source (see Supporting Information for full details of the 85 features). This label‐free, image‐based determination of eosinophil activation‐status is validated by the comparison of traditional and imaging cytometry metrics in a control cell set and the one exposed to the stimulatory agent, eotaxin 28.

## Materials and Methods

### Cell Extraction and Preparation

Human peripheral blood was collected from healthy volunteers into heparinized Vacuettes™ (Greiner Bio‐one, Frickenhausen, Germany). Blood was processed within 30 min of collection. All samples were collected with informed written consent, and ethical approval was obtained from Wales Research Ethics Committee 6 (13/WA/0190).

### Eosinophil Activation in Whole Blood

Whole blood (500 μl/tube) was exposed to a stimulus known to induce piecemeal degranulation—100 ng/ml exotoxin (CCL11)(BioLegend, San Diego, CA), for 2 or 5 h at 37°C. Untreated samples were also included. After incubation, 200 μl of blood was removed, red blood cells were lysed, and the sample was then fixed (BD CellFix™, Becton Dickinson, Belgium).

### Image Acquisition by Imaging Flow Cytometry

The sample volume for imaging flow cytometry was 50 μl. The Imagestream 100 (Merck, Darmstadt, Germany) platform was used to capture images. The Imagestream 100 provided 10,000 cell events from which eosinophils were gated postacquisition. For each cell, images of brightfield, dark field (90° to illumination), and fluorescence (488‐nm excitation, 505–560‐nm emission band) were collected. The fluorescent channel was used to identify unstained eosinophils within a blood leukocyte population as they are identified by a marked autofluorescence signal in comparison with other leukocytes 29. This signal is generated by the high concentration of flavin adenine dinucleotide localized within the cytoplasmic granules 30. After image acquisition, the IDEAS 6.0 software tool (Merck, Darmstadt, Germany) was used for initial data analysis of whole cell intensity in each of the three collection channels.

*Imaging Cytometer settings*: Sample volume: 50 μl. Flow diameter: 10 μm. Velocity of flow: 66 ms^−1^. Resolution: 1 μm. Magnification: ×40. Camera sensitivity: 32 on all channels. Camera gain: 1. Brightfield LED intensity: 36 mW. Darkfield laser intensity: 40 mW. 488‐nm laser intensity: 60 mW.

### Image Analysis

Following initial sample gating to select single, focused cells, all eosinophil cell images were exported from the IDEAS software environment for detailed analysis, as *tagged image format files*. Multivariate analysis was based on extraction of pixel‐related features in the side‐scatter image. The *Cellprofiler* open source software platform 31 was used to obtain a set of 85 image metrics reporting on signal intensity, radial distribution, image granularity, and image texture (full *Cellprofiler* pipeline available in Supporting Information). Diffusion mapping of the data set to reduce dimensionality to a 2‐D plot was implemented using an open‐source MATLAB code (Laurens van der Maaten, Delft University of Technology).

### Software Availability

The designed workflow software is open source and freely available:

Cellprofiler: http://www.cellprofiler.org/imagingflowcytometry

Multivariate analysis: https://lvdmaaten.github.io/drtoolbox/

## Results

### Whole Cell Analysis

**Figure 1:**
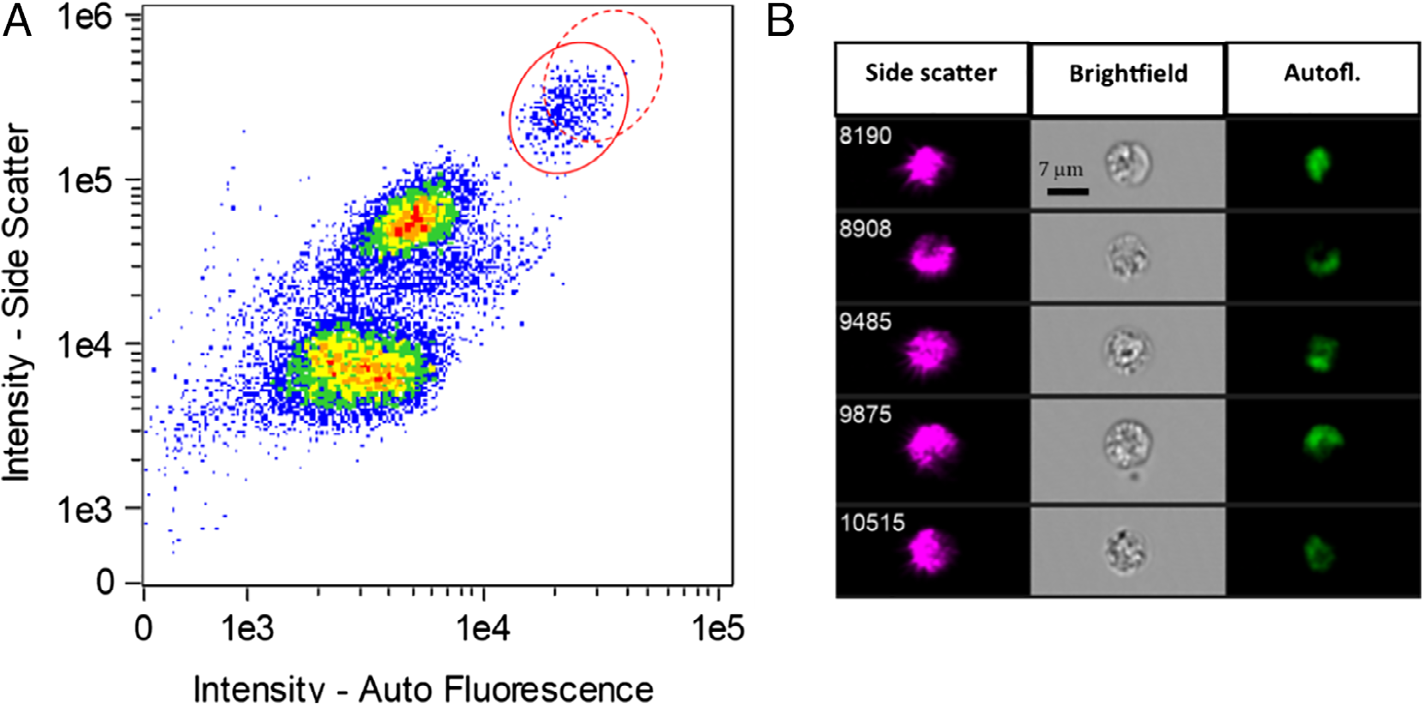
(**A**) scatter plot of side‐scatter intensity versus autofluorescence intensity for an untreated, control sample. The eosinophil population is identified by the solid red circle. The shift typically induced by exposure to an activating agent is shown by the dashed red circle. (**B**) Typical side scatter, brightfield, and autofluorescence images of five eosinophils. [Color figure can be viewed at http://wileyonlinelibrary.com]

**Figure 2:**
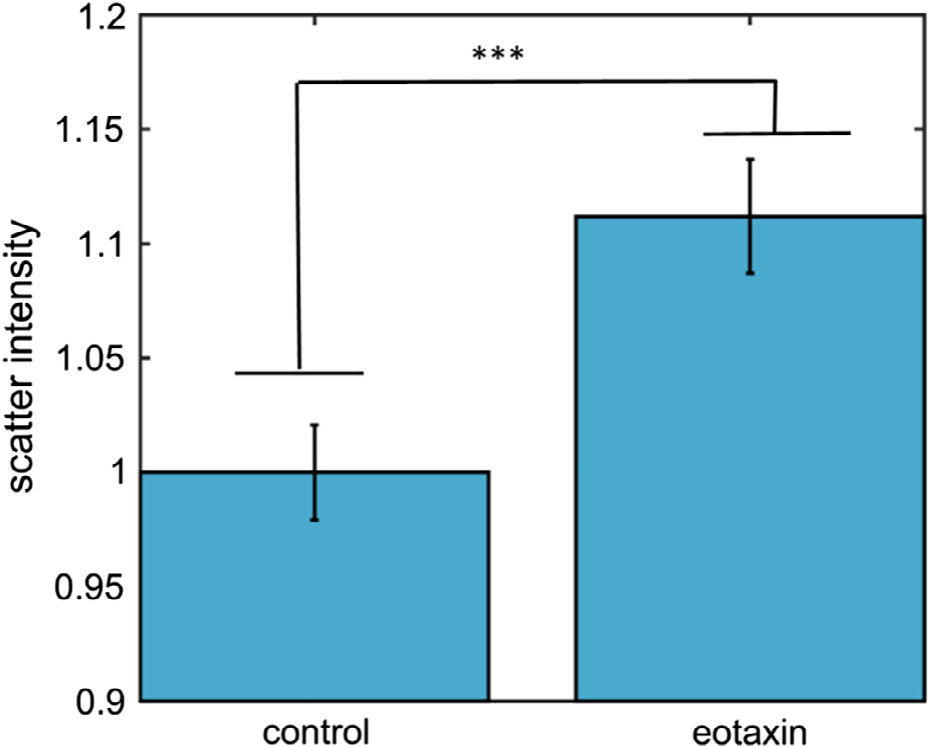
Mean side‐scatter intensity of eosinophil cell samples following a 2‐h exposure to eotaxin. Measurements are scaled to give unity value for the control (untreated) sample, error bars show the s.e.m. (*n* = 1,075, total cell count acquired from three separate measurement of the donor sample). [Color figure can be viewed at http://wileyonlinelibrary.com]

Identification of eosinophils within the leukocyte population is based on whole cell parameters of integrated autofluorescence and side‐scatter intensity. A typical scatter plot is shown in Figure 1A; the high optical scattering coefficient and fluorescent protein content of the eosinophil granules produce a clear clustering of these cells in the upper right quadrant, discriminating them from the other leukocyte subgroups (validation of the gating using CD markers is presented in Supporting information). Bright‐field images of gated eosinophils are presented in Figure 1B, together with the scatter and the autofluorescence signals. The intracellular granules appear as dark spots within the bright field (transmission mode) as the illuminating light beam is scattered away from the optical path. The other channels confirm the spatial correspondence of these dark spots to the scatter and fluorescence image maxima. To explore the link between the activation state and the optical properties of cells, samples were exposed to a known activation agent—eotaxin 17, 32—for a 2‐h period. This produced a statistically relevant increase of ~10% in the mean scatter intensity per cell (Fig. 2). Thus, the biological and morphological changes in eosinophils, which occur upon activation, clearly affect the optical properties of these cells.

### Subcellular Analysis: Multivariate Image Feature Mapping

**Figure 3:**
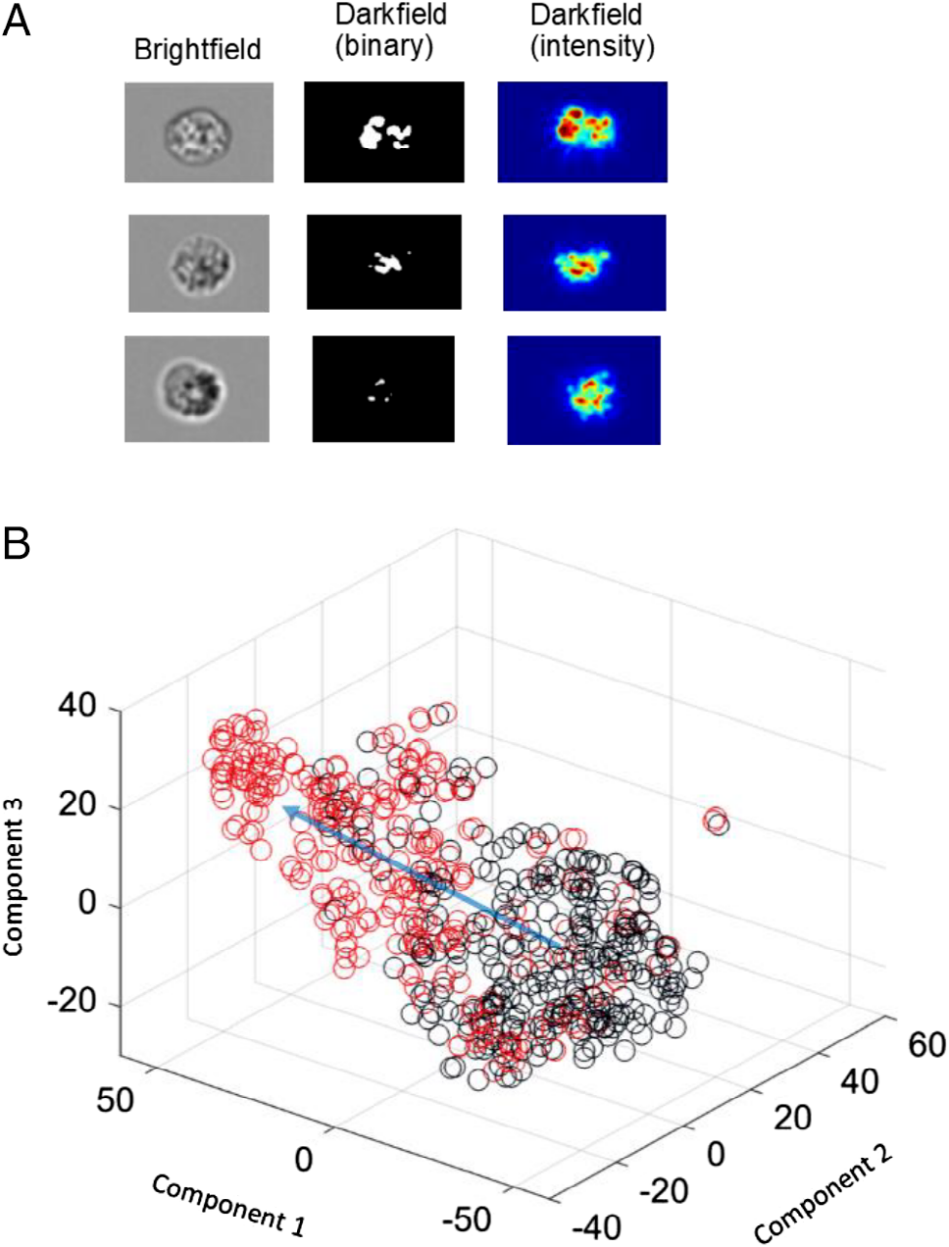
(**A**) Representative images of three cells showing brightfield; threshold‐implemented binary darkfield, in which granule morphology is evident; and darkfield intensity images, which show the texture and intensity features. (**B**) PCA plot of control (black) and eotaxin‐treated (red) samples (exposure time of 5 h). [Color figure can be viewed at http://wileyonlinelibrary.com]

Our purpose was to avoid predetermined analysis approaches using predefined feature measures, and therefore, we adopted an open search for a reporting metric based on multiple parameters, extracted from the cell images. A panel of typical images is shown in Figure 3A. The scatter channel was chosen for feature extraction, as it is less susceptible to optical phase artifacts. These appear in a subset of bright‐field images in the form of bright or dark halos around the cell 33 and are due to small deviations of cell position within the flow stream. A linear mapping of the 85 image features to a 3‐dimensional PCA plot is shown in Figure 3B. This clearly shows a shift in cell position as activation via eotaxin changes the granular attributes of individual cells. While the PCA provides good population‐level discrimination between control and activated samples, detailed analysis of single cells is limited, for this is a linear data reduction technique, and so cannot capture the full complexity of the cell trajectory within the geometry of the multivariate feature manifold 34. We therefore adopted a nonlinear, diffusion mapping approach. Diffusion mapping is a time inference or pseudotime algorithm in which the Euclidean distance separation of data points within the multivariate space is equated to a mathematical probability function 35. The manifold of point‐to‐point distances is thus captured within a probability matrix, whose eigenfunctions can be used as coordinates for low‐dimensional representation. Conceptually, the approach is well suited to the analysis of dynamic cell populations 36 as it is assumed that they “diffuse” through the multidimensional state space as their structural or functional form alters. In relation to the activation of eosinophils, it is the changes in their granular morphology that alter the image metrics and, hence, move the cell position within the multivariate space.

**Figure 4:**
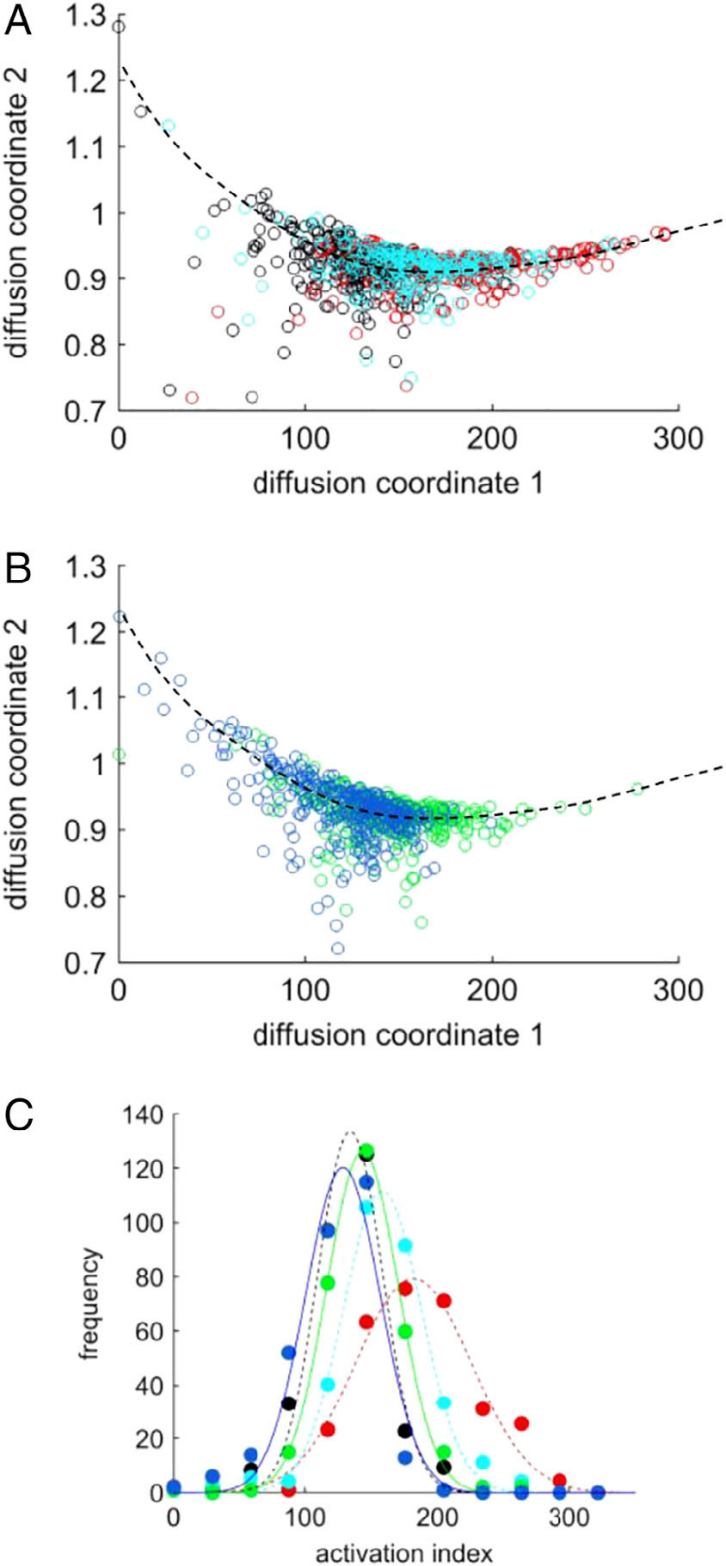
(**A**) Diffusion map of gated eosinophil populations for exogenous activation with eotaxin, 1.9% eosinophil sample (untreated control—black; 2 h exposure—cyan; 5 h exposure—red). Black dashed line is a guide to the eye of the diffusion path. (**B**) Diffusion map for endogenous activation (3.1% eosinophil sample—blue, 9.0% sample—green). [Each point corresponds to a single cell, numbers per sample vary across the set within the range: 215 < *n* < 302]. (**C**) Histograms of activation index, obtained from diffusion maps, for all five samples (color coding as per panels A and B). Points indicate histogram bin values, and lines are Gaussian‐fitting curves. [Color figure can be viewed at http://wileyonlinelibrary.com]

The data matrix for the diffusion mapping contained the combined image feature metrics from five samples: three with differing eosinophil counts (1.9%, 3.1%, and 9.0% of WBC total) and no exogenous activation, and two further samples from the 1.9% eosinophil donor, subjected to 2‐ and 5‐h eotaxin exposure. By pooling the data in this way, we maximize the occupancy of the state space and provide the diffusion‐mapping algorithm with the best possible representation of the potentiality of cell status. This also allows us to use the results of the mapping to make direct comparisons between all cells. The presentation of the results is split into two subplots to maintain clarity. The results of the diffusion mapping for eosinophils exogenously activated with eotaxin, for differing duration, are shown in Figure 4A. Cells lie along a curved trajectory within the 2‐D space with increasing eotaxin exposure producing a rightward shift of cells relative to the control sample. Given that the three data sets are from a single donor sample and have undergone a unified mapping within the multivariate space, we correlate cell position along the 2‐D trajectory of Figure 4A to the degree of activation. Statistical testing of the data, using a two‐sample, Kolmogorov–Smirnov test, indicates 99.9% probability that the three samples come from different distributions. As elevated blood eosinophil count is a biomarker of eosinophilic activity 37, the diffusion map should also differentiate samples from donors with different eosinophil numbers, that is, profile an endogenously activated cell set. The results in Figure 4B confirm that this is the case with high‐count samples sitting further along the trajectory. Thus, the position of each cell along the diffusion path, from the *y*‐intercept zero point, can be used as a measure of the relative activation status of the cell. Histograms of this “activation index” are shown in Figure 4C and are consistent with elevated levels of cell activation in samples exposed to an activation agent or in samples from donors with elevated eosinophil counts. Inspection of the relative influence of the 85, image metrics on the diffusion map shows that the 2‐D representation is primarily determined by 16 texture measurements (Haralick indices), eight measurements of intensity (e.g., mean, median, std. dev., lower quartile %), and a granularity measure.

**Figure 5:**
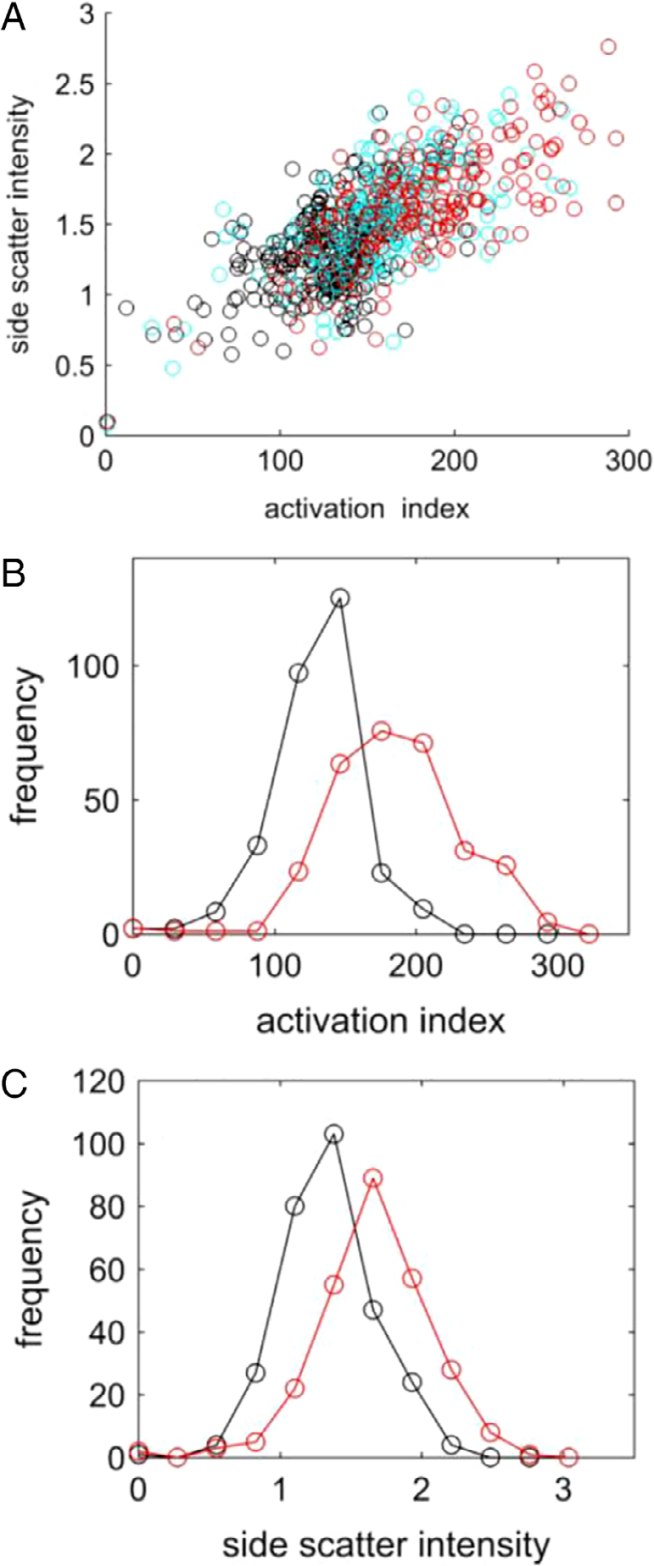
(**A**) Scatter plot of side‐scatter intensity versus activation index (untreated control—black, *r*_p_ = 0.54; 2 h exposure—cyan, *r*_p_ = 0.63; 5 h exposure—red, *r*_p_ = 0.73). (**B**) Histograms of activation index for untreated control (black) and sample exposed to eotaxin for 5 h (red). Fisher discriminant ratio, *r*_d_ = 0.70. (**C**) Histograms of side‐scatter intensity for untreated control (black) and sample exposed to eotaxin for 5 h (red), *r*_d_ = 0.47. [Color figure can be viewed at http://wileyonlinelibrary.com]

Given that whole cell intensity measures are playing some role and that mean population scatter level correlates with activation (see Fig. 2), the question may be raised as to the value of undertaking cell imaging rather than traditional flow cytometry. This is addressed in Figure 5, which shows a comparison of activation measures based on the single metric of side‐scatter intensity (integrated image intensity) and multiple, spatially resolved metrics (diffusion map position). The scatter plot in Figure 5A indicates a good correlation between the different measures for the highly activated sample (5‐h eotaxin exposure). However, the correlation is considerably weaker for the control sample data. Histograms of each metric for control and eotaxin‐exposed samples are shown in Figure 5B and C. In both cases, the null hypothesis is rejected at the *P* < 0.01 level (Kolmogorov–Smirnov test), but the greater discriminatory power of the multivariate, activation index produces a Fisher discriminant ratio, *r*_d_ of 0.7 compared with a value of 0.47 for side‐scatter intensity alone. These results indicate that for highly activated cells, where the optical scattering is pronounced, side‐scatter intensity can be used as a reasonable surrogate of activation status. In less‐activated cells, however, the scattering is not enough to give an unambiguous indication of cell activation, and in this case, the additional information content provided by image analysis leads to greater measurement resolution.

## Discussion

The aim of this study was to identify cell position within a continuous activation status profile for granular leukocytes. Related examples can be found in work reporting on the quantitative tracking of cells through the continuum of the cell cycle, either by nonlinear data clustering 3 or using deep learning in neural networks 4. We employed diffusion mapping as this technique provides a means to track arbitrary movement within a multivariate space. The diffusion map produces Euclidean distances in an embedded space, which approximate to diffusion distances in the data, and has proven effective in analyzing evolution of cell state due to biological process, for example, stem‐cell differentiation pathways 36, 38. The analysis presented here on blood eosinophils confirms that scatter‐image features due to intracellular granule content and distribution can be identified using imaging cytometry and that diffusion mapping of these does indeed allow quantitative profiling of the activation status of cells across a population. This approach therefore adds an additional diagnostic tool, over and above measurement of eosinophil counts, and the technique is sensitive enough to distinguish between activation histograms from donor samples in which the eosinophil count differs by just 1%.

Integration of signals from the microenvironment preferentially activates different secretory pathways within eosinophils; discrete activation profiles measurable by image analysis of peripheral blood eosinophils could therefore provide insight into disease phenotype 39 or endotype 37 and response to treatment. Allergic disease, asthma, and parasite infection have been the focus for eosinophil‐related research these past decades, but there is growing appreciation of their contribution to host defense against bacteria and viruses, various hypereosinophilic syndromes, autoimmune disease, cancer, and a range of eosinophilic gastrointestinal disorders that are increasing in prevalence 40. There is a real need for noninvasive methods of diagnosing these eosinophilic gastrointestinal disorders and for biomarkers for multiple eosinophilic diseases. As an example, eosinophilic esophagitis is a disease that has emerged in the past 30 years, is associated with food allergy, afflicts children and adults, and can be diagnosed only by symptoms and histology of esophageal biopsy 41. More nuanced phenotyping of peripheral blood eosinophil activation status might limit the need for invasive endoscopy for tissue sampling, especially in children.

## Supporting information


**Figure SI** 1: scatter plot of mean side‐scatter intensity versus mean autofluorescence intensity. The sub‐population indicated in red was positive for a CD193(CCR3) antibody label.
