# Supplementary material for: Diffusion Mapping of Eosinophil‐Activation State
Source: Cytometry A. 2019 Aug 31;97(3):253–8. doi: 10.1002/cyto.a.23884 (PMC7079009; doi:10.1002/cyto.a.23884)
Supplement: Supplementary file 1 — Figure SI 1: scatter plot of mean side‐scatter intensity versus mean autofluorescence intensity. The sub‐population indicated in red was positive for a CD193(CCR3) antibody label. [file CYTO-97-253-s001.docx]

**SUPPLEMENTARY INFORMATION**

**Diffusion mapping of Eosinophil activation state using image features**

*J. Piasecka, C.A. Thornton, P. Rees and H.D. Summers*

**SI 1: Identification of eosinophils**

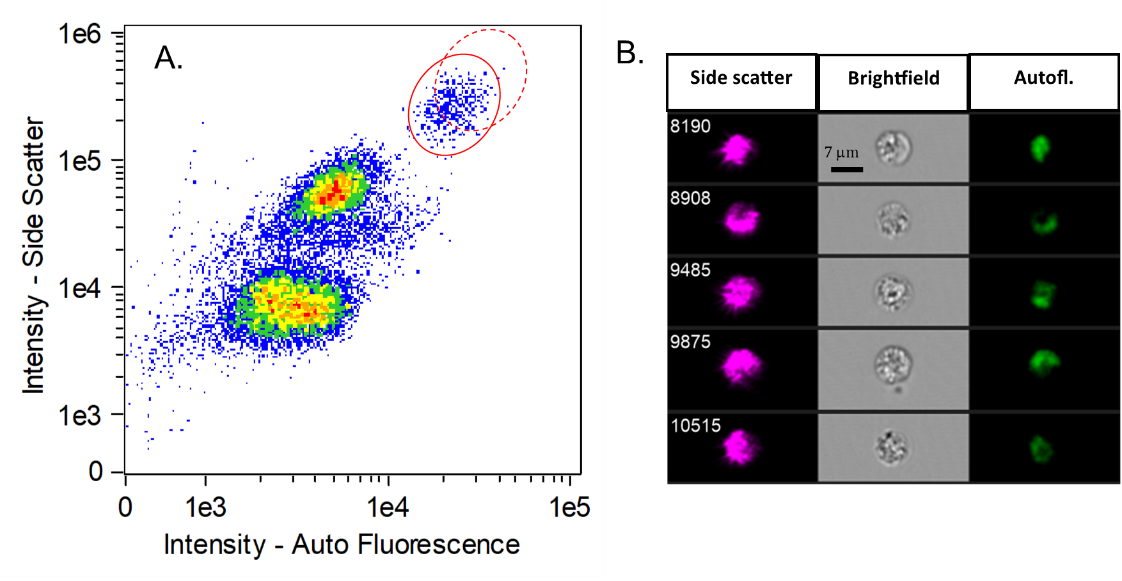


*Figure 1A from main manuscript.*

*Figure SI 1: scatter plot of mean side-scatter intensity versus mean autofluorescence intensity. The sub-population indicated in red was positive for a CD193(CCR3) antibody label.*

Cells were labelled with a known eosinophil marker – CD193(CCR3). The CD193 +’ve population corresponds to the top-right hand sub-population (+’ve AF, +’ve SSC), thus validating the eosinophil gating procedure shown in figure1A of the manuscript (shown alongside).

**SI 2: Cell Profiler analysis pipeline**

Individual cell images are exported from the IDEAS software platform in *.tiff* format. To reduce file handling individual images are then tiled into 9x9 arrays and saved as a new meta-image, this is done with custom software using MATLAB. The meta-images, each containing 81 cells are processed using the Cell Profiler software platform. The Cell Profiler pipeline is shown below; following image read, each cell is identified as a primary object by a thresholding algorithm. Feature metrics are then extracted for granularity, intensity, distribution, texture and shape.


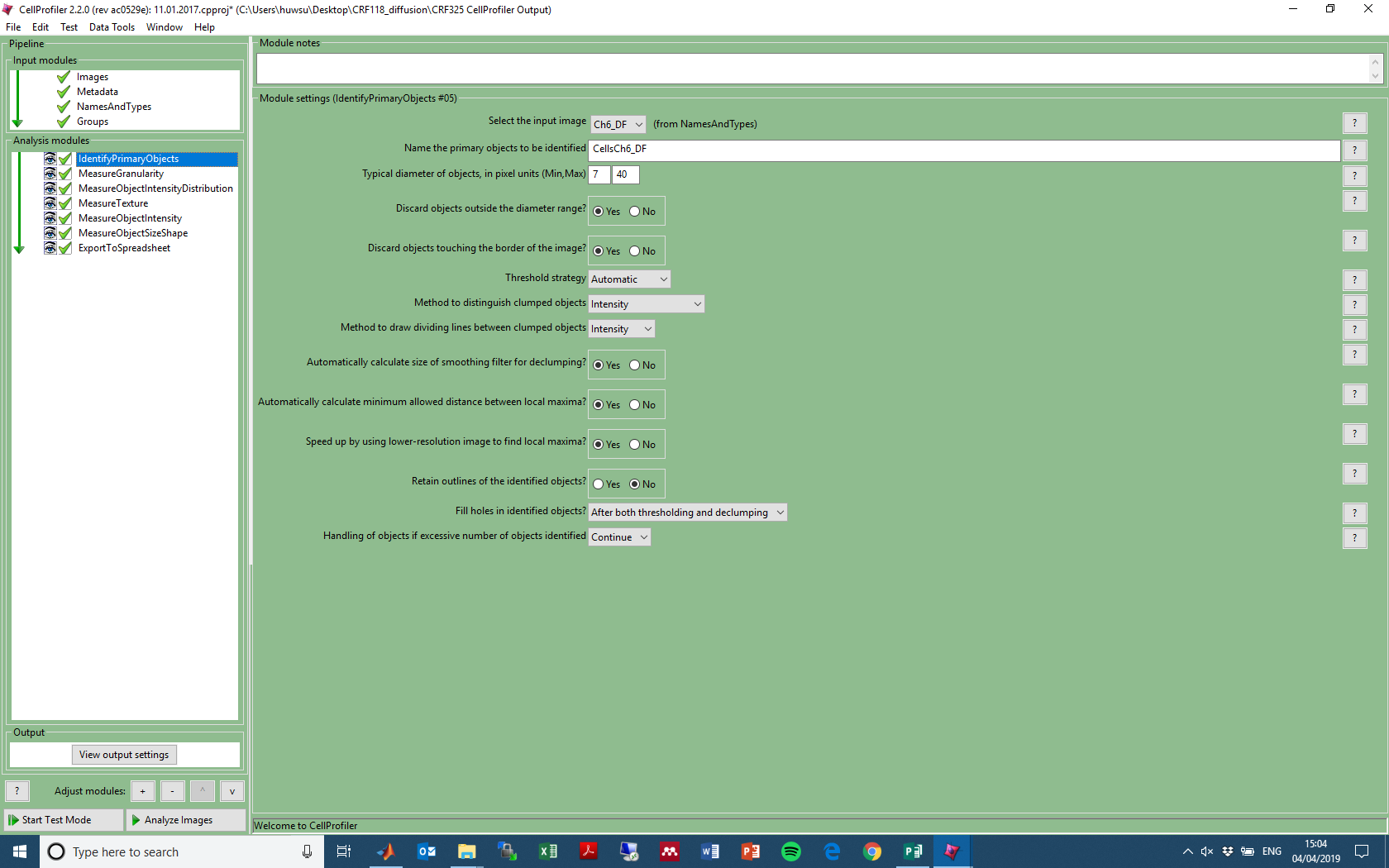


**SI 3: Image features**

The full list of 85 metrics extracted from each cell image is as follows -

***Granularity:***

Granularity_1_DF, Granularity_2_DF, Granularity_3_DF, Granularity_4_DF, Granularity_5_DF

***Intensity:***

Intensity_IntegratedIntensityEdge_DF, Intensity_IntegratedIntensity_DF, Intensity_LowerQuartileIntensity_DF, Intensity_MADIntensity_DF, Intensity_MassDisplacement_DF, Intensity_MaxIntensityEdge_DF, Intensity_MaxIntensity_DF, Intensity_MeanIntensityEdge_DF, Intensity_MeanIntensity_DF, Intensity_MedianIntensity_DF, Intensity_MinIntensityEdge_DF, Intensity_MinIntensity_DF, Intensity_StdIntensityEdge_DF, Intensity_StdIntensity_DF, Intensity_UpperQuartileIntensity_DF

***Radial distribution:***

RadialDistribution_FracAtD_DF_1of4, RadialDistribution_FracAtD_DF_2of4, RadialDistribution_FracAtD_DF_3of4, RadialDistribution_FracAtD_DF_4of4, RadialDistribution_MeanFrac_DF_1of4, RadialDistribution_MeanFrac_DF_2of4, RadialDistribution_MeanFrac_DF_3of4, RadialDistribution_MeanFrac_DF_4of4, RadialDistribution_RadialCV_DF_1of4, RadialDistribution_RadialCV_DF_2of4, RadialDistribution_RadialCV_DF_3of4, RadialDistribution_RadialCV_DF_4of4

***Texture:***

Texture_AngularSecondMoment_DF_3_0, Texture_AngularSecondMoment_DF_3_135, Texture_AngularSecondMoment_DF_3_45, Texture_AngularSecondMoment_DF_3_90, Texture_Contrast_DF_3_0, Texture_Contrast_DF_3_135 ,Texture_Contrast_DF_3_45, Texture_Contrast_DF_3_90, Texture_Correlation_DF_3_0, Texture_Correlation_DF_3_135, Texture_Correlation_DF_3_45 Texture_Correlation_DF_3_90, Texture_DifferenceEntropy_DF_3_0, Texture_DifferenceEntropy_DF_3_135, Texture_DifferenceEntropy_DF_3_45, Texture_DifferenceEntropy_DF_3_90, Texture_DifferenceVariance_DF_3_0, Texture_DifferenceVariance_DF_3_135, Texture_DifferenceVariance_DF_3_45, Texture_DifferenceVariance_DF_3_90, Texture_Entropy_DF_3_0, Texture_Entropy_DF_3_135, Texture_Entropy_DF_3_45, Texture_Entropy_DF_3_90, Texture_Gabor_DF_3, Texture_InfoMeas1_DF_3_0, Texture_InfoMeas1_DF_3_135, Texture_InfoMeas1_DF_3_45, Texture_InfoMeas1_DF_3_90, Texture_InfoMeas2_DF_3_0, Texture_InfoMeas2_DF_3_135, Texture_InfoMeas2_DF_3_45, Texture_InfoMeas2_DF_3_90, Texture_InverseDifferenceMoment_DF_3_0, Texture_InverseDifferenceMoment_DF_3_135, Texture_InverseDifferenceMoment_DF_3_45, Texture_InverseDifferenceMoment_DF_3_90, Texture_SumAverage_DF_3_0, Texture_SumAverage_DF_3_135, Texture_SumAverage_DF_3_45, Texture_SumAverage_DF_3_90, Texture_SumEntropy_DF_3_0, Texture_SumEntropy_DF_3_135, Texture_SumEntropy_DF_3_45 Texture_SumEntropy_DF_3_90, Texture_SumVariance_DF_3_0, Texture_SumVariance_DF_3_135, Texture_SumVariance_DF_3_45, Texture_SumVariance_DF_3_90, Texture_Variance_DF_3_0, Texture_Variance_DF_3_135, Texture_Variance_DF_3_45, Texture_Variance_DF_3_90
